# Compounded Complexity: The Impact of Social Determinants of Health on a Child With Cerebral Palsy and Lennox-Gastaut Syndrome

**DOI:** 10.7759/cureus.108247

**Published:** 2026-05-04

**Authors:** Nayade Caldes, Nicole M Perez, Juliana Quintero, Katherine Semidey

**Affiliations:** 1 Pediatrics, Nova Southeastern University Dr. Kiran C. Patel College of Osteopathic Medicine, Davie, USA; 2 Pediatrics, Florida International University Herbert Wertheim College of Medicine, Miami, USA

**Keywords:** lennox-gastaut syndrome, low-income patients, social determinant, student-run free clinic, uninsured patients

## Abstract

Cerebral palsy (CP) is a common motor disorder in children; however, when combined with Lennox-Gastaut syndrome (LGS), it presents as a rare and highly complex condition requiring coordinated, multidisciplinary care. This case report highlights the challenges faced by a recently immigrated, uninsured child with limited financial resources, demonstrating how these barriers contributed to the progressive deterioration of her condition. It shows the significant impact of social determinants of health (SDoH) on the well-being and survival of children with complex medical needs. Addressing these challenges requires increased awareness of available resources and stronger collaboration among healthcare providers to improve access to comprehensive, continuous care for vulnerable populations.

## Introduction

Cerebral palsy (CP) is one of the most common motor disorders in children, affecting approximately 1 in 345 children in the United States, with higher prevalence and severity in low- and middle-income countries [1,2]. Children with CP often experience delayed developmental milestones, abnormal muscle tone, and impaired motor coordination [3].

Nearly half of children with CP also develop epilepsy; of those, 1%-2% are diagnosed with Lennox-Gastaut syndrome (LGS), a severe, treatment-resistant epileptic encephalopathy (disorder affecting brain function). The combination of CP and LGS creates significant clinical complexity, requiring intensive, multidisciplinary management [4]. Without coordinated care, these children face worsening disability and life-threatening complications.

For low-income children who are ineligible for free or low-cost insurance, access to such care is often limited or entirely unavailable. Social determinants of health (SDoH) are the conditions in which people are born, grow, live, work, and age, factors that can either support health and well-being or create barriers to care and recovery. In a multitude of ways, SDoH plays a critical role in influencing the outcomes of children with CP and LGS. Factors such as financial instability, limited access to comprehensive healthcare, inadequate transportation, food insecurity, and a lack of social and familial support create significant barriers to consistent and effective treatments. These disparities lead to delayed diagnoses, reduced access to medications, specialist care, and therapies, frequent hospitalizations, and poorer overall prognosis in vulnerable populations [5].

Despite the recognized need for coordinated care, access to such services remains uneven, particularly among underserved populations. This case report describes a 13-year-old uninsured immigrant child with CP and LGS who died from complications of aspiration pneumonia. It highlights how gaps in access to multidisciplinary care, driven by structural and socioeconomic inequities, contributed to her rapid decline. We also describe how our free clinic, serving low-income and uninsured families, attempted to address some of these barriers through coordinated, community-based support.

## Case presentation

Consent was obtained from the patient and family for the following case presentation.

A 13-year-old, uninsured, recently immigrated female from Nicaragua with cerebral palsy (CP) and Lennox-Gastaut syndrome (LGS) presented to our free clinic in April 2024 with her mother for post-hospital discharge follow-up and to establish ongoing care. Her mother expressed significant concern regarding the need for coordinated follow-up with multiple subspecialists following her daughter’s recent hospitalization.

Medical history and barriers before immigration

According to her mother, the patient experienced her first visible seizure at eight years of age and was subsequently evaluated at a clinic in Nicaragua, where she was diagnosed with cerebral palsy and epilepsy. The family lived in a rural, resource-limited setting with significant financial constraints, limiting access to consistent healthcare services. In the months following diagnosis, visible seizure frequency increased to approximately one to two episodes per week.

The patient initially received physical therapy through Teleton, a nonprofit organization, but due to Nicaragua’s sociopolitical instability, securing appointments became exceedingly difficult, limiting the patient to a single physical therapy session every three months. Neurology appointments were particularly difficult to access, requiring a three-hour journey to the capital city and a significant financial burden. In the absence of reliable transportation and continuous general pediatric care, these arduous trips served as the family’s only means of obtaining the patient’s medications and refills.

Facing overwhelming obstacles while their daughter’s condition worsened, the family immigrated to the United States seeking improved access to medical care, resources, and long-term support. Following immigration, the patient remained uninsured, and the family relied on prescriptions obtained during well-child visits to secure anti-seizure medications through the Epilepsy Foundation. Due to the absence of consistent neurology follow-up, medication dosages were not adjusted during this period. Additionally, rescue medications for acute seizure management were unaffordable and therefore unattainable.

First US hospitalization: initial diagnosis and the clinic’s first intervention

Two years after immigration, the patient presented to the emergency department with fever, respiratory distress, and clinically observed seizures occurring approximately 15 days apart. She was admitted to the pediatric intensive care unit (PICU) for respiratory distress and status epilepticus secondary to aspiration pneumonia with a large pleural effusion. During this admission, she also underwent a swallow study where her silent tracheal aspiration was confirmed, followed by placement of a gastrostomy tube (GT) with Nissen fundoplication.

Once discharged, the patient was placed on a strict GT diet consisting of Boost nutritional shakes. Her new medication regimen included oxcarbazepine, glycopyrrolate, lansoprazole, and valproic acid by GT. Upon discharge, she was instructed to follow up with her pediatrician, physical therapist, speech therapist, gastroenterologist, neurologist, dentist, and pulmonologist. Information for our free clinic was provided by the social worker at that time. Although the patient was granted emergency Medicaid in order to cover the costs of her inpatient hospitalization, it did not extend to outpatient specialty care, preventing her from accessing the recommended follow-up care.

Initial Clinic Visit and Social Barriers

Within one week of discharge, the patient presented to our free clinic for post-hospital follow-up and to establish care. Her mother expressed concerns about affording medications, formula, GT supplies, and coordinating multiple specialty appointments.

On examination, the patient was severely underweight, wheelchair-bound with spasticity and poor coordination, minimally verbal, and dependent on a diaper due to severe immobility. She required GT feeds administered while lifted onto the clinic bed. She lived in a single-room household with her parents and younger sibling, with her father serving as the sole income provider and her mother as the primary caregiver. Significant financial strain, transportation limitations, and language barriers were reported, as the mother did not drive and required language interpretation during medical encounters, and the father’s demanding work schedule limited appointment availability.

In response, the clinic connected the family with nonprofit organizations to offset medication costs. The Epilepsy Alliance Foundation and Miami Pediatric Fund provided coverage for antiepileptic medications and agents for secretion and gastrointestinal management. A volunteer surgical nurse practitioner conducted a telemedicine visit to evaluate postoperative GT care. Through charitable organizations, the patient received one hour of physical therapy weekly for a limited period; however, speech therapy services remained inaccessible. The clinic also successfully enrolled the patient in the county Special Transportation Services due to her disability.

A single gastroenterology consultation was obtained, during which botulinum toxin injections were recommended. However, ongoing access to neurology, pulmonology, nutrition, respiratory therapy, and continued gastroenterology care remained financially unattainable.

Subsequent hospitalizations and clinic aid

In the months that followed, the patient continued to experience copious oral secretions and a persistent cough. Despite multiple medication adjustments coordinated by the clinic’s pediatrician in private consultation with pulmonology and gastroenterology teams from her prior hospitalization, her symptoms remained refractory. Neuromuscular impairment related to cerebral palsy, complicated by her worsening oral-motor dyscoordination due to her progressive LGS encephalopathy, significantly limited effective secretion management. This resulted in recurrent aspiration events and aspiration pneumonia with subsequent hospitalizations.

Following her third hospitalization, the patient demonstrated progressive clinical worsening, with increasing seizure burden and evolution to more severe episodes characterized by respiratory compromise. Precise quantification was limited, as caregivers were often unable to recognize seizure activity until advanced, clinically apparent events occurred. Within one month of discharge, she was rehospitalized for aspiration pneumonia requiring prolonged PICU admission and intubation for respiratory failure. Subsequent hospitalizations increased in frequency, duration, and acuity, often necessitating mechanical ventilation and escalating interventions to manage refractory seizures and secretion burden.

During the patient’s fourth and fifth hospitalizations, interventions to manage secretions progressed from medical therapy to more invasive measures, including botulinum toxin injections to parotid and submandibular glands, transdermal scopolamine, and ultimately salivary gland ablation, with limited sustained benefit. Following discharge each time, outpatient subspecialty management depended on continued remote coordination with hospital-based consultants volunteering their time. During this period of escalating illness, our clinic fundraised to support the family’s growing medical needs, including formula, medications, and essential supplies. Despite continued efforts by the clinic to bridge gaps through care coordination, fundraising, and resource navigation, the cumulative burden of her illness intensified, placing increasing emotional, financial, and logistical strain on the family.

Her final hospitalization occurred in June 2025 following a prolonged seizure with aspiration. She presented in respiratory distress, developed acute kidney failure, and was transitioned to palliative care. She passed away peacefully, surrounded by family.

This case illustrates the devastating consequences of fragmented care in medically complex, socially vulnerable pediatric patients, and highlights both the critical role and inherent limitations of safety-net clinics working to bridge these gaps.

## Discussion

This case illustrates the multifactorial challenges our team encountered while caring for a pediatric patient with comorbid cerebral palsy (CP) and Lennox-Gastaut syndrome (LGS), further complicated by significant financial insecurity, lack of transportation and social support, and absence of health insurance. These barriers critically limited her access to consistent care, specialized services, and life-sustaining medications.

**Figure 1:**
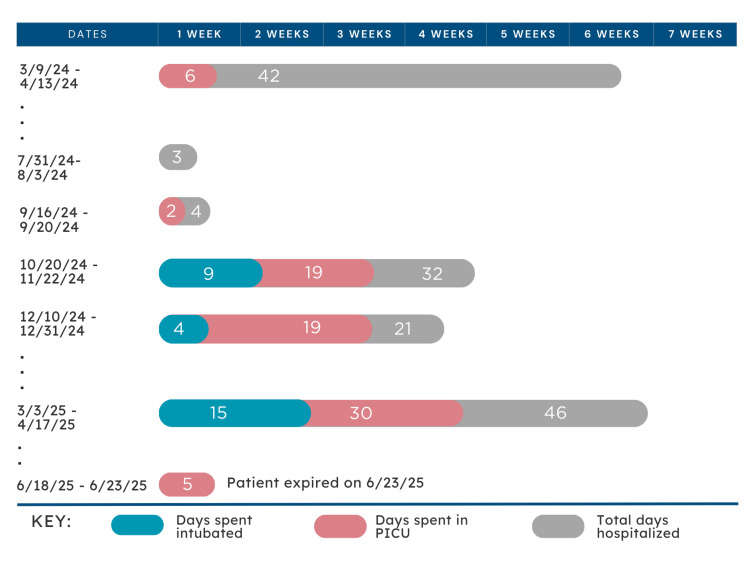
Hospitalization Course Highlighting PICU and Intubation Durations This figure illustrates the patient’s recurrent hospitalizations over time, including total days hospitalized (gray), days spent in the PICU (pink), and days requiring intubation (blue). Hospitalizations increased in frequency and duration over the clinical course, culminating in the final admission in June 2025, during which the patient expired. PICU: pediatric intensive care unit

Lennox-Gastaut syndrome (LGS) is a rare and severe form of epilepsy that typically begins in early childhood and presents with a complex array of medical challenges that profoundly impact quality of life. Children with LGS often experience multiple, frequent seizure types that are highly resistant to standard anti-seizure medications [6]. These seizures are not only difficult to manage but also often debilitating, worsening over time, and contributing significantly to the overall burden of the disease. With each clinically observed seizure, the patient exhibited a visible and distressing decline in her physical, cognitive, and emotional state; however, given the high likelihood of frequent subclinical seizures unnoticed by her mother, the true burden of her neurologic decline was likely even greater. This is reflected in Figure 1, showing a progressive increase in the frequency of hospitalizations, with each admission associated with longer PICU stays and prolonged intubation durations. Although LGS follows a known trajectory of deterioration, the patient’s socioeconomic context likely accelerated disease progression and complicated management.

The social determinants of health played an undeniable and personal role in worsening her outcomes. Our patient lived in a small room with her mother, father, and younger sister, where her complex medical needs occupied a significant portion of the household’s physical and emotional space. Her mother stayed home full-time to provide around-the-clock care, including monitoring seizures, managing secretions, administering medications and feedings, and assisting with all aspects of daily living. Meanwhile, her father worked long hours as a construction worker, battling the immense burden of being the sole provider for their family while navigating the emotional weight of his daughter’s condition. Despite long, arduous work hours, he only earned $1,000 per month to support his family of four.

Prior literature has shown that caregivers of children with medical complexity experience substantial out-of-pocket expenses related to their child’s care, contributing to a significant financial burden [7]. In this case, and as shown in Table 1, estimated monthly costs for medications, nutritional support, and essential medical supplies totaled approximately $1,035, surpassing the family’s total monthly income. As a result, consistent access to medications, therapies, and subspecialty follow-up was not feasible, contributing to fragmented care and increased hospitalization frequency.

**Table 1:** Monthly Cost Breakdown of Medication, Supplies, and Nutritional Support

Category	Item	Monthly Cost (USD)
Respiratory Care	Albuterol	15.27
Respiratory Care	Sodium Chloride	5.97
Respiratory Care	Ipratropium	16.25
Seizure Management	Valproic Acid	51.37
Seizure Management	Oxcarbazepine	50.00
Seizure Management	Diazepam Rescue	93.60
Secretion Control	Glycopyrrolate	86.15
Secretion Control	Scopolamine Patches	43.18
Gastrointestinal Nutrition	Lansoprazole	187.30
Gastrointestinal Nutrition	Boost Plus 30 days	330.00
Gastrointestinal Nutrition	Poly-Vi-Sol	15.99
Medical Supplies	Suction Tubes	8.00
Medical Supplies	Size 10 Deep Catheter	88.00
Daily Care Needs	Diapers	40.00
Daily Care Needs	Wipes	4.00
Total Monthly Cost	-	1,035.08

During extended hospitalizations, the mother faced the painful challenge of splitting her time and attention between her hospitalized child and her younger daughter at home. This would lead the family to face chronic stress, emotional exhaustion, and a lack of social support, which contributed to signs of maternal depression and placed significant strain on the parents’ relationship.

These challenges were intensified by the family’s lack of support. Without nearby relatives or a support network, they faced their daughter’s complex medical needs largely on their own. School became the patient’s only source of social connection outside her immediate family. According to her mother, she was well-loved by classmates, and her mood noticeably brightened during school days. However, as her condition deteriorated, it became increasingly unsafe for her to remain in such a high-risk environment, leading her mother to make the difficult decision to withdraw her from school. This marked the loss of the last source of social engagement and sense of normalcy in the patient’s life. The lack of community and extended family support left the family feeling isolated within a healthcare system that was often overwhelming and difficult to navigate.

**Figure 2:**
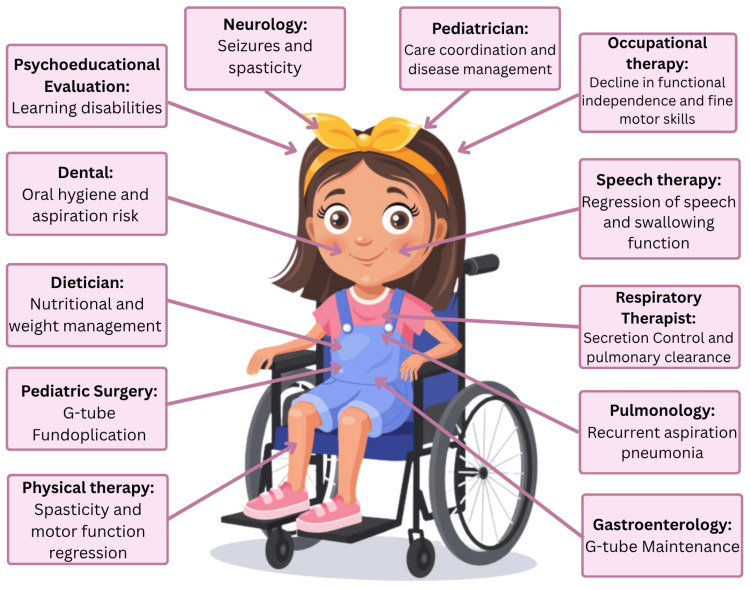
Multidisciplinary Care Needs for This Patient With CP, LGS, and Aspiration Pneumonia The figure depicts the coordinated services required for comprehensive management, including neurology, pulmonology, gastroenterology, nutrition, speech and swallowing therapy, respiratory therapy, physical therapy, and social services. In this case, access to multiple services was delayed, fragmented, or unavailable due to social determinants of health. CP: cerebral palsy, LGS: Lennox-Gastaut syndrome Illustration adapted from an image purchased from iStock (iStock by Getty Images) and used under a standard royalty-free license

Figure 2 illustrates the multidisciplinary services required to meet the complex medical needs of our patient with Lennox-Gastaut syndrome (LGS), cerebral palsy (CP), and aspiration pneumonia. Unfortunately, this level of coordinated care was not available to her. While living in Nicaragua, she encountered major barriers to care, including inconsistent access to speech therapy, physical therapy, and specialty testing and care. These obstacles were driven by systemic issues such as inadequate national funding for rehabilitative care and limited transportation infrastructure, which made it difficult for her family to attend clinical visits. After immigrating to the United States, our patient’s situation remained precarious. She did not qualify for health insurance and, as a result, continued to lack access to essential multidisciplinary care, which was now compounded by language barriers, financial insecurity, and fear of living undocumented.

While there are no standardized treatment guidelines for managing cerebral palsy (CP) and Lennox-Gastaut syndrome (LGS) concurrently, each condition independently requires coordinated, multidisciplinary care involving neurologic, rehabilitative, and supportive services [8,9]. Despite the collective efforts of local nonprofits, a pediatric interest group within our local medical school, and our free clinic, we were unable to secure the full scope of resources our patient needed for her complex diagnoses. As an uninsured child from a low-income, non-English-speaking household living with both cerebral palsy (CP) and Lennox-Gastaut syndrome (LGS), her care presented profound and multifaceted challenges. A recent neurology study found that Hispanic patients are 40% less likely to receive outpatient neurology care, suggesting that factors such as racial bias, clinical uncertainty, and stereotyping may contribute to this disparity [10]. In our patient’s case, limited English proficiency was one of the many challenges her family faced. Financial hardship, lack of transportation, and absence of social support further complicated her access to care, affordability of services, and comprehension of essential medical information. Together with inaccessibility of essential services such as physical and speech therapy, specialist care for optimized treatment, and proper multidisciplinary care to effectively manage her complex factors, all remained heartbreakingly out of reach.

## Conclusions

This case emphasizes the challenges faced by a medically complex pediatric patient lacking consistent access to multidisciplinary care. The patient’s clinical course, marked by recurrent hospitalizations, progressive respiratory complications, and increasing care needs, occurred in the context of significant socioeconomic barriers, including lack of insurance, financial constraints, and limited access to specialty services. These factors were associated with fragmented care and limited the ability to provide consistent outpatient management. Despite efforts from a free clinic and community-based organizations to support medication access, nutrition, and care coordination, gaps in subspecialty care and rehabilitative services persisted. This case illustrates the difficulty of managing complex neurologic conditions in resource-limited settings and highlights how barriers to care may impact clinical trajectory. While this report describes a single patient, it underscores the importance of addressing access to coordinated care for children with complex medical needs and the role of safety-net systems in supporting vulnerable populations.
